# Transparent and Water-Resistant Composites Prepared from Acrylic Resins ABPE-10 and Acetylated Nanofibrillated Cellulose as Flexible Organic Light-Emitting Device Substrate

**DOI:** 10.3390/nano8090648

**Published:** 2018-08-23

**Authors:** Xueping Song, Shuang Yang, Xiuyu Liu, Min Wu, Yao Li, Shuangfei Wang

**Affiliations:** 1 Department of Pulping and Papermaking Engineering, College of Light Industry and Food Engineering, Guangxi University, Nanning 530004, China; sx_ping@gxu.edu.cn (X.S.); 1616391011@mail.gxu.cn (S.Y.); xiuyu.liu@wmich.edu (X.L.); wumin@gxu.edu.cn (M.W.); 2 Guangxi Key Laboratory of Clean Pulp & Papermaking and Pollution Control, Guangxi University, Nanning 530004, China; 3 Department of Chemical and Paper Engineering, Western Michigan University, Kalamazoo, MI 49008-5200, USA; 4 Department of Pulping and Papermaking Engineering, Guangxi Vocation & Technical Institute of Industry, Nanning 530004, China; jiangyan306@mail.gxu.cn

**Keywords:** acetylated nanofibrillated cellulose, acrylic resins ABPE-10, composite films, flexible organic light-emitting device substrate, interpenetrating polymer network

## Abstract

Acetylated nanofibrillated cellulose (ANFC)/acrylic resin ABPE-10 composite film was prepared by impregnating ABPE-10 into ANFC films under negative pressure, which can enhance properties of ANFC films by forming an interpenetrating polymer network structure between ABPE-10 and the ANFC film. The ANFC/ABPE-10 composite film met the high performance flexible organic light-emitting diode substrate requirement, even when the ANFC dosage was as high as approximately 70%. The transparency of films with different ANFC dosages significantly increased from 67% (42 µm) to 88% (45 µm), as determined by ultraviolet-visible analysis. The composite film inherited the properties of AFNC, with a low coefficient of thermal expansion and a ductile compact structure. The contact angles of ANFC films increased from 49.2° to 102.9° after dipping in ABPE-10. Additionally, the composite films had good surface smoothness and mechanical properties.

## 1. Introduction

Organic light-emitting diodes (OLEDs) have unique features, including a wide viewing angle, high efficiency, low power consumption, and high response speed, and are inexpensive, which has enabled the development highly portable and flexible OLEDs [1,2]. Flexible OLEDs (FOLEDs) typically include an anode, hole injecting layer, hole transporting layer, organic emitting layer, electron transporting layer, electron injection layer, cathode, and organic material films, as well as at least one transparent electrode to create a shiny surface, which are all the important for a FOLED device [3,4,5]. FOLEDs have been applied to a wide range of fields, such as information, energy, healthcare, and defense fields, due to their flexibility, high efficiency, and low fabrication costs [6,7,8,9]. FOLED displays will have a profound impact on the application of wearable and portable devices, and will be widely used with the continuous development of personal intelligent terminals [10,11,12,13]. With the growing sophistication of organic light-emitting materials and device technologies, FOLEDs are regarded as promising technology for future displays [6,14].

FOLEDs are fabricated based on and operated in a flexible substrate, so the substrate is an important part of flexible device. The substrate must provide mechanical support and simultaneously process photonic and electronic information for the FOLED equipment. Thus, the quality of the substrate determines the performance and life expectancy of the device. The FOLED substrate should be smooth and flat, with good dimensional stability, high flexibility, good thermal stability, high transparency, and good barrier properties [14]. Additionally, substrate materials impact the following processes: electrode deposition, barrier coating, patterning exposure, and thin-film transistor fabrication. Therefore, selecting the proper substrate materials is critical. In current FOLED substrates, some synthetic soft polymer matrices are expected to replace the glass substrate, but their large coefficient of thermal expansion (CTE) limits their use.

Cellulose is an abundant renewable raw material [15]. Nanofibrillated cellulose (NFC) is prepared from natural fibers via mechanical grinding and is composed of linear microfibril aggregates with higher aspect ratio. NFC has the tendency of self-linking through hydrogen bonding interaction between fibers, and the three-dimensional rigid network can be formed within a matrix [16]. NFC is flexible, non-friable, lightweight (density 1.5 g/cm^3^), and biodegradable, with low CTE (8 × 10^−6^ K), large surface area (>50 m^2^/g), and excellent mechanical properties compared with conventional glass materials. In addition, the thin film prepared from NFC is transparent and strong [17], so it can potentially be applied in high technology fields as a conductive NFC film [18] and electronic substrate [19]. When the amount of added NFC is low (≤5%), NFC composites, such as FOLED substrates, still have superior thermal stability, mechanical and barrier properties, and recyclability [20]. In addition, the sheet-by-sheet processing of the FOLED substrate can be replaced by large-scale roll-to-roll processing [21]. Therefore, the cost-efficient and environmentally friendly FOLED substrate produced by NFC is attracting intensive research and commercial interest.

Our previous studies found that acetylated nanofibrillated cellulose (ANFC) films, such as FOLED substrates, have many advantageous properties, including a smooth surface, high flexibility, and good thermal and mechanical properties. However, the light transmittance of NFC films was about 70%, and had not reached the 80% requirement for FOLED substrates [14]. Its water resistance also needed to be enhanced. Acrylic resin ABPE-10 is colorless, transparent and water-insoluble. Compared with the traditional heat-cure technology, light-cure technology is faster, more efficient, and has better mechanical properties, while curing could take place at room temperature [22]. Also, the costs of light-cure equipment and energy consumption are low, so acrylic resins ABPE-10 is known as an environmentally friendly green material. Okahisa et al. [19] impregnated an NFC film in acrylic resins and tetrahydrocyclopentadiene dimethacrylate, and obtained a composite material with high transparency. Nogi and Yano et al. [23,24] prepared a flexible substrate by compositing bacterial-cellulose nanofibers films and acrylic resins. The nanocomposites performed well in terms optical properties, dimensional stability, and thermal performance. However, these studies rarely describe the relationship between ANFC film and acrylic resin ABPE-10, and have rarely focused on improving the transparency of NFC films produced by mechanically ground NFC for preparing the highly transparent FOLED substrates. Furthermore, the combination of ANFC film and ABPE-10 to improve the water resistance of ANFC membranes as FOLED substrates has rarely been reported.

**Figure 1:**
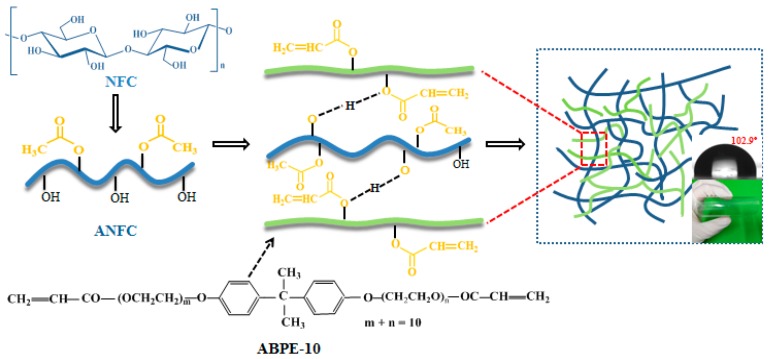
Interpenetrating polymer network (IPN) of acetylated nanofibrillated cellulose (ANFC)/acrylic resin ABPE-10 composite film.

ABPE-10 contains carboxylic acid ester and a phenyl structure, as an amphiphilic substance (Figure 1). The carboxylic acid ester structure of ABPE-10 can form hydrogen bonds with the hydroxyl groups (–OH) in the NFC, enhancing the binding between NFC film and ABPE-10, and improving the evenness of ABPE-10 on the surface of an NFC film. After drying, the phenyl structure of ABPE-10 can also improve the oxidation resistance and water resistance of the FOLED substrate. Additionally, acrylic resin ABPE-10 contains double functional groups (–CH=CH_2_), which can accelerate ultraviolet (UV) light polymerization speed. The three-dimensional (3D) network structure is composed of an ANFC network and an ABPE-10 network, which is partially interlaced on the molecular scale by hydrogen bonding, but is not covalently bonded to each other. Thus, the structure of the ANFC/ABPE-10 composite film should be an interpenetrating polymer network (IPN). The interaction mechanism of ANFC film and ABPE-10 in composite materials is shown in Figure 1.

In this study, in order to further improve the light performance, smoothness, and water resistance of FOLED substrates based on ANFC, we produced the ANFC/ABPE-10 composites with high ANFC content by simulating the papermaking process. Moreover, the combination relationship between ANFC film and ABPE-10 was illustrated, also the transmittance and water resistance of ANFC film as FOLED substrate were improved.

## 2. Materials and Methods

### 2.1. Materials

Bleached softwood kraft pulp (*Pinus khasys*), provided by Yun-Jiang Forestry & Pulp Mill Co., Ltd. (Yunnan, China), containing 96.90% cellulose, 3.50% hemicellulose, and less than 0.1% lignin, was used as the raw material. 1-hydroxycyclohexyl phenyl ketone was used as the photo initiator, 2-hydroxyethyl acrylate (analytically pure) as the reactive diluent, and 2,2-bis[4-(acryloxy polyethoxy) phenyl] propane (acrylic resin ABPE-10) were obtained from Shin Nakamura Chemical Co., Ltd. (Tokyo, Japan). All reagents (Aladdin Co., Ltd., Shanghai, China) were analytically pure.

### 2.2. Preparation of ANFC

The 3 wt % pulp was ground through a grinder (Super Masscolloider MKZA 10-15JIV, Masuko Sangyo Co., Ltd., Saitama, Japan) at 1,500 rpm for 30 min. After grinding, the pulp was diluted with distilled water to 0.2 wt % pulp suspension and passed through a high-pressure homogenizer (GJJ-0.06/40; Keju Fluid Equipment Manufacturing Co., Ltd., Shanghai, China). The homogenization conditions were as follows: 2 times at 0 bars, 3 times at 400 bars, and 3 times at 600 bars.

The NFC suspension was replaced repeatedly with acetone through vacuum filtration in order to obtain the NFC acetone suspension. Similarly, the NFC toluene suspension was obtained. NFC acetylation was conducted by placing 25 mL toluene, 20 mL acetic acid, and 0.1 mL perchloric acid into the NFC (83 wt %, 1.0 g dry) in sequence. Then, 3 mL acetic anhydride was added to the NFC and was stirred continuously for 1 hour at room temperature. After acetylation, the ANFC was washed thoroughly with ethanol and distilled water by centrifugal separation (8 min each time, 10,000 rpm/min), respectively, until the pH of the filtrate reached 7.0 measured by a pH meter.

In order to avoid the environmental pollution, the solvent and acid used in the process of acetylation was recovered using nanofiltration and solvent extraction technology [25,26].

### 2.3. Preparation of ANFC Films

The ANFC slurry (0.2, 0.3, 0.4, 0.5, and 0.6 g bone dry) was diluted to 0.2 wt %. The suspension of diluted ANFC was stirred for 2 hours to ensure its dispersion. Then, the dispersed ANFC was vacuum filtered with G2 sand core funnel (90 mm diameter), which was padded with a layer of hydrophilic polyterafluoroethylene organic filter membrane (0.22 µm pore size, 90 mm diameter) in advance. The wet ANFC film was removed together with the organic filter membrane after filtering, covering another organic filter membrane on the other side of ANFC film. The filter papers were covered on the surface of the organic filtering films before drying in order to speed up the removal of moisture. The wet ANFC film was pressed from both sides with glass in order to obtain a flat film during drying. The film was then dried at room temperature for 12 h, and moved to a vacuum drying oven at 55 °C for 24 h.

### 2.4. Preparation of IPN ANFC/ABPE Composite Films

The ANFC films were impregnated in acrylic resin ABPE-10 at a pressure of −0.09 MPa for 12 h. ANFC films were removed after impregnating, and a small coating machine was used to scrape the extra ABPE-10 on the surface. Then the films were solidified for 3 min under a 1000 W UV light using a light-cure machine (BDS-2000, Shenzhen, China) with the main peak 365 nm of spectra, and the ANFC/ABPE composite films were obtained. At last, the films were balanced more than 24 h under 25 °C and 50% humidity.

### 2.5. Analysis

#### 2.5.1. Transmission Electron Microscopy (TEM)

The morphology, dimension, and yield of NFC and ANFC were studied by TEM at 100 kV (TECNAI G2 F30, Hillsboro, OR, USA). The NFC and ANFC suspension was diluted to 0.01 wt %, and dispersed for 30 min with ultrasonic waves. A small amount of diluted dispersion was carefully dropped on a 200 mesh carbon-coated grid. After drying at room temperature, the sample was dyed with phosphotungstic acid stain for 20 min. The morphology distribution and particle size of the samples were analyzed using the Nano Measurer 1.2.5 software (Fudan University, China).

#### 2.5.2. Determination of Acetylation Degree

The degree of substitution (DS) of samples was measured by proton nuclear magnetic resonance (^1^H-NMR) spectroscopy (Bruker AC III HD600, Germany) [27]. ^1^H-NMR spectra were measured with an aspectrometer using tetramethylsilane as the internal standard at 256 scanning repetitions and 500 MHz. The sample was dissolved in dimethyl sulfoxide (DMSO-d6), and the DS was calculated according to Goodlertt [28].

#### 2.5.3. Optical Properties

Using a single lens reflex (SLR) Nikon d7100 camera (Nikon Corporation, Yokohama, Japan, Japan) the apparent transmittances of films were photographed in a well-lit laboratory. A UV-Vis Spectrometer Lambda 950 (PerkinElmer, Waltham, MA, USA) was used to measure the light transmittance of films in visible wavelength range from 380 nm to 780 nm. The samples were cut into 10 × 10 mm^2^ pieces and placed 25 cm from the outlet of the integral sphere.

#### 2.5.4. Apparent Morphology

Atomic force microscopy (AFM, Hitachi High-Technologies Corporation 5100N, Chiyoda Ward, Tokyo, Japan) was used to characterize the surface roughness and morphological stability of films (10 × 10 mm^2^) with tapping mode. AFM probes type is SI-DF40P2, f = 299 KHz, C = 32 N/m, and tip diameter is 7 nm. The average roughness values of the films were determined from a 1 µm^2^ area of three images per sample, and were presented as an average root-mean-square (rms) value. The apparent morphology and tensile fracture-surfaces of the samples were sprayed with gold, and were then observed by field emission scanning electron microscopy at 10.0 kV (FE-SEM, Hitachi High-Technologies Corporation SU 8020, Chiyoda Ward, Tokyo, Japan). The ANFC/ABPE-10 composite film (10 × 10 mm^2^) folded to −180° and +180° were observed by scanning electron microscopy (SEM, Phenom F16502, Eindhoven, Netherlands) at 10.0 kV.

#### 2.5.5. Contact Angle

The contact angles were measured using a contact angle meter (DSA100, Hamburg, Germany). The specimens were 40 mm long and 10 mm side. A droplet of distilled water was deposited on the flat film. The contact angle was measured on 3 different points and the average values were calculated.

#### 2.5.6. Thermal Performance

The film CTE was measured by a thermomechanical analyzer (Q400, TA Instruments, New castle, PA, USA). The measurement conditions were as follows: specimens 25 × 3 mm^2^, pull 0.03 N, temperature increased from 30 °C to 150 °C with a heating rate of 5 °C/min. The test was conducted under nitrogen conditions, and each sample was circulated three times. The CTE values were determined by the average value of the second run and the third run in order to eliminate the residual stress of the membrane material. CTE values are provided as the average and standard deviation (error bars) of three independent determinations for each sample.

#### 2.5.7. Mechanical Properties

The Young’s modulus, tensile strength, and elongation at break of the samples were measured using a Shimadzu AG-X testing machine (Nakagyo Ward, Kyoto, Japan). The specimens were 25 mm long and 3 mm wide. The measurement conditions were as follows: load sensor 50 N, gauge length 20 mm, and stretching rate 1 mm/min. Three test samples were measured and the data were reported as an average and standard deviation (error bars) of tests.

## 3. Results and Discussion

### 3.1. Characteristic Analysis of ANFC

**Figure 2:**
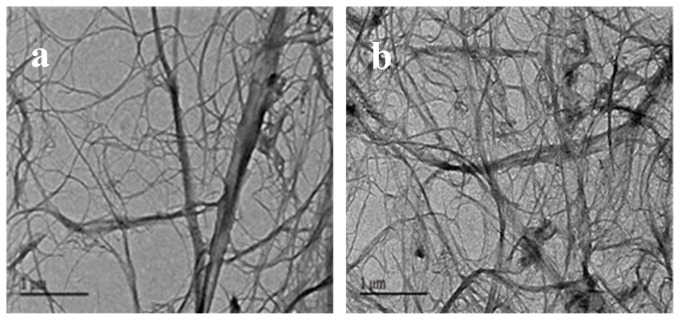
Transmission electron microscopy (TEM) images of (**a**) nanofibrillated cellulose (NFC) and (**b**) acetylated nanofibrillated cellulose (ANFC).

TEM micrographs of NFC and ANFC are displayed in Figure 2, and both NFC and ANFC had a great aspect ratio. The NFC and ANFC dimensions were obtained by measuring at least 100 individuals from the TEM images. From Figure 2a, more than 80% of the diameter of individual NFC was estimated to be within the range of 5 to 30 nm. Approximately 80% of the diameter of ANFC had an average between 5 and 20 nm (Figure 2b). The acetylation had little effect on fiber dimension, which was similar to the finding of Jonoobi et al. [29]. By analyzing the ^1^H-NMR spectra in Figure S1, the emergence of methyl hydrogen signals at 1.8–2.1 ppm in Figure S1b, which indicated that C2 hydroxyl groups were replaced by acetyl groups. In our earlier study, ANFC film had the best performance when the DS of ANFC was 0.24, and a more detailed analysis was provided in our previous work [14].

### 3.2. Effects of Different ANFC Dosages on the Optical Properties of Composite Films

Transparency is the most crucial property for bottom emissive displays; a FOLED substrate must have an 80% total light transmission of in the visible light range of 380 to 780 nm [30,31]. The composition and thickness of the composite films are described in Table 1.

**Figure 3:**
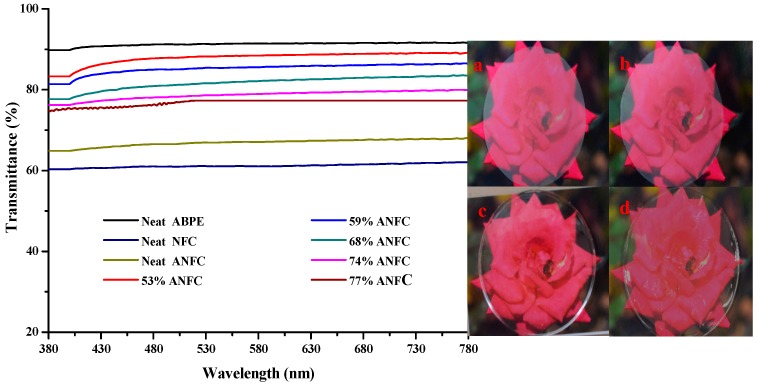
The effects of different ANFC dosages on the transparency of the samples, and the apparent photographs of (**a**) neat NFC film, (**b**) neat ANFC film, (**c**) neat ABPE-10 film, and (**d**) ANFC/ABPE-10 composite film.

The transparency of the samples (neat ANFC film, neat ABPE-10 film, and ANFC/ABPE-10 composite films was illustrated by photographing and UV-Vis Spectrometer analysis (Figure 3). Since the ANFC/ABPE-10 composite films with different ANFC dosages had similar images, only the ANFC/ABPE-10 composite film with an ANFC dosage of 68% is presented as an example here. From Figure 3, although the bee on the red rose of background image could be displayed through these films, the transparency of neat NFC film and neat ANFC film was lower than that of neat ABPE-10 film and ANFC/ABPE-10 composite film. The transparency of neat ABPE-10 film was slightly higher than that of ANFC/ABPE-10 film.

As shown in Figure 3, the transparency of neat NFC film was the lowest compared to the other films, followed by the neat ANFC film, indicating that acetylation could slightly improve the transparency of films. The transparency of the ANFC/ABPE-10 composite films with different ANFC dosages, compared with that of neat ANFC film, significantly increased from 67% to 88% at a wavelength of 600 nm at about 45 µm thickness, indicating that the combination of ABPE-10 and ANFC improved the transparency of ANFC film. The main reason for this finding was that the ABPE-10 filled the gaps in the ANFC film and decreased the surface roughness of the film. The rms value is crucial for the roughness of the film, which is the mean of the root for the height deviation from the standard surface to the indicated surface [32]. The average rms value of ANFC film obviously reduced from 11.010 nm to 2.865 nm after being impregnated with ABPE-10 from the Figure S2. The average rms value of ANFC/ABPE-10 composite film was nearly the same as that of neat ABPE-10 film (see detail in the Figure S2), which indicated that the ABPE-10 on the surface of composite film could effectively reduce the roughness of film and suppressed the scattering of photons, resulting in a low scattering index [24].

**Figure 4:**
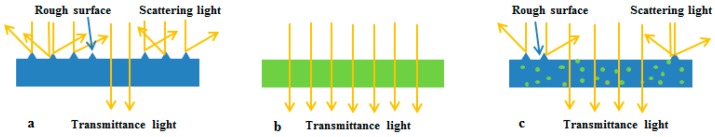
The light scattering path of films: (**a**) neat ANFC film, (**b**) neat ABPE-10 film, and (**c**) ANFC/ABPE-10 composite film (68% ANFC).

Mimicking of the light scattering paths on different surfaces of films are shown in Figure 4. With the increase in the ANFC dosage in the composite film, the transparency of ANFC/ABPE-10 composite films decreased by varying degrees. This occurred because the dense network structure in films was formed through hydrogen bonding between fibers, which prevented further impregnating with acrylic resin ABPE-10, as shown in Table 1. The results mean that ABPE-10 did not completely fill the surface porosity of ANFC film and decreased its surface smoothness, further causing different degrees of light scattering. Meanwhile, the increase in ANFC dosage led to a slight increase in the thickness of films (Table 1), eventually also causing a decrease in the composite film transparency. However, when the ANFC dosage in the composite film was less than 68%, the transparency of ANFC/ABPE-10 composite film was maintained at 80%, meeting the FOLED substrate requirement. Okahisa at al. found comparable transparency when the ANFC dosage was lower, around 35–40% [19].

### 3.3. Effects of Different ANFC Dosages on the Apparent Qualities of Composite Films

**Figure 5:**
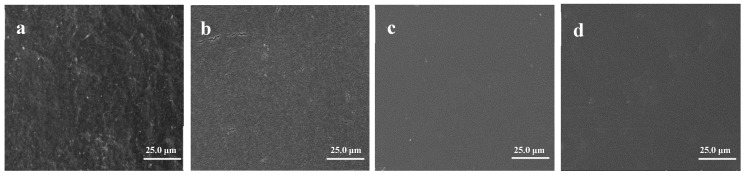

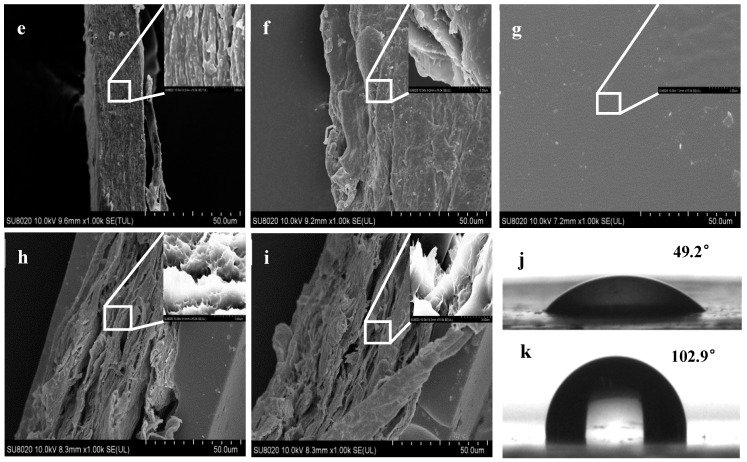
The apparent images of samples: (**a**) neat NFC film, (**b**) neat ANFC film, (**c**) neat ABPE-10 film, and (**d**) ANFC/ABPE-10 composite film (68% ANFC). The tensile cross-section images: (**e**) neat NFC film, (**f**) neat ANFC film, (**g**) neat ABPE-10 film, (**h**) NFC/ABPE-10 composite film, and (**i**) ANFC/ABPE-10 composite film (68% ANFC). The contact angle images of: (**j**) neat ANFC film and (**k**) ANFC/ABPE-10 composite film (68% ANFC).

Some surface qualities, including roughness, cracks, and cleanliness, are important to guarantee the integrity of subsequent barrier and conductive layers. Cracks in a substrate may lead to the formation of pinholes on the thin films of the electrode, creating dark spots in OLEDs. Also, the cracks would be serious when the displays are bent. In order to determine the apparent morphology, stable performance, and bonding degree of ANFC and ABPE-10, both the apparent morphology and tensile cross-section of films were analyzed by FE-SEM. Since the apparent morphology, tensile cross-section, and contact angle of ANFC/ABPE-10 composite films with different ANFC dosages had similar results, only the ANFC/ABPE-10 composite film with 68% ANFC dosage is presented as an example here. The results are shown in Figure 5.

From Figure 5, the surface of neat ABPE-10 film was both smoothest and cleanest, followed by the ANFC/ABPE-10 composite film, under the same magnification of 1.0 K. Also, some small cracks were observed on the surface of neat ANFC film. Compared with neat ANFC film, the surface of ANFC/ABPE-10 composite film was smoother, and the surface crack had been largely filled, but the surface smoothness was slightly lower than the neat ABPE-10 film. Based on these results, we concluded that acrylic resin ABPE-10 could fill the gaps in ANFC film and improve the smoothness of ANFC film. The results further confirmed that acrylic resin ABPE-10 improved the transmittance of the composite film, which was consistent with the observation from Figure 3.

By recording the tensile cross-section of neat ABPE-10 film, we found that the cross-section was smooth and flat. Also, the fracture direction of the cross-section was consistent with an obvious rigid structure. Under external load, the ability of such materials to resist being torn and bent was limited, leading to rapid expansion of cracks according to the propagation direction of the cracks. These materials presented a typical brittle fracture [33]. Thus, neat ABPE-10 film was not suitable as a FOLED substrate. Comparing with the neat NFC film image, neat ANFC film had a fracture surface that was separated into flakes, scales, and layers, which proved that the fracture was a result of ductile tearing and the ANFC film was tougher material. Fortunately, the composite films prepared by brittle ABPE-10 and ductile NFC/ANFC presented obvious ductile tearing, as shown in Figure 5h,i, which was due to the fiber having outstanding properties in terms of good expansivity, superior flexibility [34], abundant hydrogen bonds, and a 3D network structure of the NFC itself, delaying the breakage. The results demonstrated the excellent strength performance of these materials as FOLED substrate. However, some holes appeared on the cross-section of NFC/ABPE-10 composite film, as shown in Figure 5h, indicating that the binding of unmodified NFC with ABPE-10 was slightly inferior compared with that of the ANFC/ABPE-10 composite film (Figure 5i). The compact structure, flakes, and wiredrawing shape were more obvious in the ANFC/ABPE-10 composite film than in Figure 5h, indicating that acetylation improved the compatibility of NFC and ABPE-10. Furthermore, the structure of the composites (Figure 5i) depicted the IPN structure and highlighted the good interaction and compatibility with the matrix. This structure has often been used in the preparation of nanocellulose-based IPN hydrogels with good moisture stability and mechanical performance [35]. The ductile compact structure of this substrate would greatly reduce the formation of pinholes on the thin films of the electrode and strengthen the bending performance of displays.

The acrylic resin ABPE-10 also significantly improved the water resistance of the ANFC film. The contact angle of the ANFC film increased from 49.2° to 102.9° after dipping in ABPE-10, as shown in Figure 5j,k. The higher water resistance of the ANFC/ABPE-10 composite film was due to superior hydrophobic property of acrylic resin ABPE-10. Acrylic resin ABPE-10 also covered the surface of ANFC film and filled the gaps between nanofibers, which reduced the hydrophilicity of cellulose by decreasing the exposure of surface hydroxyl. Moreover, the capillary effect of the fiber surface was severely weakened as a result of the decrease in the microtubes of the fiber itself and the interfiber pore. We venture to guess that these results should improve the gas barrier property of ANFC film.

### 3.4. Effects of Different ANFC Dosages on the Thermal Performance of Composite Films

The thermal stability of the substrate is also important. Improved dimensional stability of composites is desired for FOLED substrates given its variable temperature during application and use. A low CTE is also beneficial when creating dimensionally stable designs for devices, and the CTE of FOLED substrate should be less than 20 ppm·K^−1^ [19,30]. Therefore, CTE of the samples were evaluated using a thermomechanical analyzer (TMA). The results are shown in Table 2.

As shown in Table 2, the CTE of neat ANFC film was about 5.43 ppm·K^−1^, and the thermal stability was better than that of neat NFC film. The CTE of neat ABPE-10 film was extremely high at 128.40 ppm·K^−1^, which would lead to unstable dimensions during application. However, the CTE of the ANFC/ABPE-10 composite films was considerably lower compared to neat ABPE-10 film, ranging from 128 ppm·K^−1^ to 11 ppm·K^−1^, which was comparable to glass [36]. Simultaneously, when the dosage of ANFC in ANFC/ABPE-10 composite film was increased from 53% to 77%, the CTE of the composite films slightly decreased. These results indicated that the dosage of ANFC with low CTE characteristics played an important role in the CTE decrease of composite films. This occurred because the thermal stability of the composites mainly depended on the thermal properties of the material and the enhancer [30]. Additionally, the 3D network structure of composite films was strengthened with the increase in ANFC weight percentage, further causing variation in composite density [37], thus limiting the thermal expansion of the composite films.

### 3.5. Effects of Different ANFC Dosages on the Mechanical Properties of Composite Films

**Figure 6:**
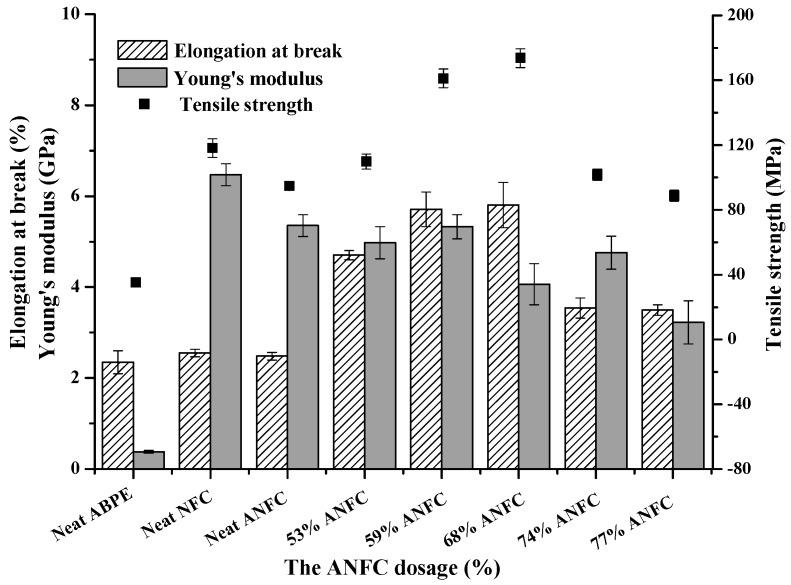
Effects of different ANFC dosages on the mechanical properties of different films.

**Figure 7:**
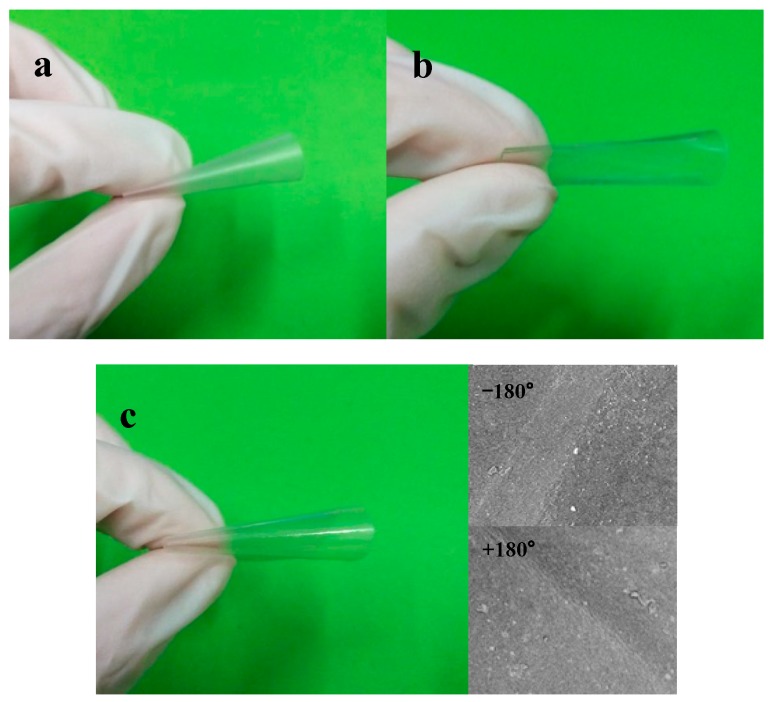
Flexibility of (**a**) neat ANFC film, (**b**) neat ABPE-10 film, (**c**) ANFC/ABPE-10 composite film (68% ANFC) and the scanning electron microscopy (SEM) images of ANFC/ABPE-10 composite film (68% ANFC) folded to −180° and +180°.

The high mechanical properties of FOLED substrate are important for maintaining the roughness and flexibility of FOLED, and meeting the roll-to-roll preparation requirement [38]. Figure 6 and Figure 7 show the mechanical properties and flexibility of different films, respectively [14,39].

The ultimate strength of composite materials, especially nanocomposites, mainly depends on the nature and volume fraction of the component and the adhesiveness and compatibility of polymer matrix and additives, as reported by Dufresne [40]. As shown in Figure 6, compared with the neat ABPE-10 film, the mechanical properties of ANFC/ABPE-10 composite films improved dramatically. When the ANFC dosage increased to 68%, the tensile strength, Young’s modulus, and elongation at break of the composite film increased 3.94, 9.68, and 1.47 fold, respectively. Compared with neat ANFC film, both tensile strength and elongation at break of ANFC/ABPE-10 composite films significantly improved. This was mainly due to the formation of an IPN structure between fibers and ABPE-10 strengthened the stability of the composite films. Importantly, those tiny pores and cracks on the surface of the ANFC film were filled by ABPE-10 when the film was impregnated in ABPE-10 under a certain pressure, enhancing the compactness of the film matrix, and thus improving its mechanical properties. However, when the ANFC dosage was more than 68%, the mechanical properties of composite films decreased sharply. One of main reasons for this decrease was that the cracks and holes on the surface of ANFC film could not be covered with the reduced adhesiveness of ANFC and ABPE-10 with the decreasing of ABPE-10 dosage in the composite film (Table 1). This eventually resulted in non-uniform force and deteriorated mechanical properties. Additionally, both the neat ANFC film and ANFC/ABPE-10 composite film presented outstanding flexibility, as shown in Figure 7. In contrast, the flexibility of neat ABPE-10 film was poor, and the film was broke. The ANFC/ABPE-10 composite film folded to −180° and +180° showed excellent flexibility without fracture on the surface of composite film, as shown in the SEM images in Figure 7. Notably, this was also supported by the apparent morphology and tensile cross-section of films shown in Figure 5.

### 3.6. Performance Comparison of Different Polymer FOLED Substrates

When the ANFC dosage was about 68% in composite film, the performance of the FOLED substrate mentioned above was maximized: transmittance 82.53%, contact angle 102.9, CTE 13.26 ppm/K, tensile strength 173.72 MPa, Young’s modulus 4.06 GPa, elongation at break 5.81%, and good flexibility. From Table 3, the CTE of polyethylene terephthalate was much larger than that of the other polymers and was not suitable for substrate requirements. The transmittance values of all polymer FOLED substrates at a wavelength of 600 nm were nearly the same. Meanwhile, the uniform deformation or stable deformation of BC (bacterial cellulose)/epoxy resin composite film was poor due to its low elongation at break. Therefore, the mechanical properties of the ANFC/ABPE-10 composite films were excellent compared to the BC/PU (polyurethane) and BC/epoxy resin composite film in Table 3. Despite the CTE of ANFC/ABPE-10 composite films being higher than the BC/epoxy resin composite film, the CTE was less than 20 ppm·K^−1^ meeting the substrate requirements. Compared with flexible nanopaper, the mechanical properties and transmittance of ANFC/ABPE-10 composite film were remarkable. The ANFC/ABPE-10 composite films prepared in this study have the following advantages: low cost, superior thermal stability, great flexibility, good biodegradability, high transparency, good smoothness, as well excellent mechanical performance, which could be combined with indium tin oxide or silver nanowires as a transparent electrode of high performance FOLED substrate.

## 4. Conclusions

An ANFC/ABPE-10 composite film was prepared by impregnating ABPE-10 into ANFC films under negative pressure. The maximized performance of the thin composite film (45 µm) met the FOLED substrate requirement when the ANFC dosage was approximately 70%. The properties of ANFC films were enhanced mainly due to the nature of ABPE-10 itself and the IPN structure formed between ABPE-10 and ANFC film. In fact, the composite films displayed high transparency (up to 80%), low CTE (13.26 ppm·K^−1^), good water resistance, and surface smoothness. Additionally, the composite films had outstanding mechanical properties including a tensile strength of 173.72 MPa, a Young’s modulus of 4.06 GPa, and an elongation at break of 5.81%. Therefore, we suggest that ANFC/ABPE-10 composite film is a potential candidate as a FOLED substrate.

## Figures and Tables

**Table 1:** The composition and thickness of the films.

Film	NFC (%)	ANFC (%)	ABPE (%)	Thickness (µm)
Neat ABPE	-	-	100	42
Neat NFC	100	-	-	42
Neat ANFC	-	100	-	42
ANFC/ABPE	-	53	47	42
ANFC/ABPE	-	59	41	42
ANFC/ABPE	-	68	32	45
ANFC/ABPE				
ANFC/ABPE				
ANFC/ABPE	-	74	26	47
ANFC/ABPE	-	77	23	48

**Table 2:** The effects of different acetylated nanofibrillated cellulose (ANFC) dosages on the coefficient of thermal expansion (CTE) of different films.

ANFC Dosage	CTE (ppm·K^−1^)
Neat ABPE-10	128.40 ± 2.47
Neat NFC	15.05 ± 0.68
Neat ANFC	5.43 ± 0.35
53% ANFC	15.32 ± 1.10
59% ANFC	15.37 ± 0.75
68% ANFC	13.26 ± 0.58
74% ANFC	11.25 ± 0.48
77% ANFC	10.91 ± 0.50

**Table 3:** Performance comparison of different polymers as flexible Organic light-emitting diodes (FOLED) substrates [24,41,42,43].

OLED Substrates	Tensile Strength (MPa)	Young’s Modulus (GPa)	Elongation at Break (%)	Transmittance (%)	CTE (ppm·K^−1^)
ANFC/ABPE-10 composite film	173.72	4.06	5.81	82.53	13.26
BC/PU composite film	69.50	6.00	1.90	82.00	-
BC/epoxy resin composite film	325.00	20.00	0.02	84.00	6.00
polyethylene terephthalate	-	2.00-2.70	7.90	83.00	20.00–100.00
flexible nanopaper	25.10	0.71	-	73.00	6.39

## Supplementary Materials

The following are available online at http://www.mdpi.com/2079-4991/8/9/648/s1, Figure S1: The proton nuclear magnetic resonance (^1^H-NMR) spectra of (a) NFC and (b) ANFC, Figure S2: Surface roughness and morphological stability of neat ANFC film, Neat ABPE-10 film, and ANFC/ABPE-10 composite film (68% ANFC).
