# Supplementary material for: Transparent and Water-Resistant Composites Prepared from Acrylic Resins ABPE-10 and Acetylated Nanofibrillated Cellulose as Flexible Organic Light-Emitting Device Substrate
Source: Nanomaterials (Basel). 2018 Aug 23;8(9):648. doi: 10.3390/nano8090648 (PMC6163516; doi:10.3390/nano8090648)
Supplement: Supplementary file 1 [file nanomaterials-08-00648-s001.pdf]

Electronic supplementary information

Transparent and Water-Resistant Composites Prepared from Acrylic Resins ABPE-10 and Acetylated Nanofibrillated Cellulose as Flexible Organic Light-emitting Device Substrate

Xueping Song [1,2,†], Shuang Yang [1,2,†], Xiuyu Liu [1,3], Min Wu [1,2], Yao Li [4], Shuangfei Wang [1,2,*]

1 Department of Pulping and Papermaking Engineering, College of Light Industry and Food Engineering, Guangxi University, Nanning 530004, China; sx_ping@gxu.edu.cn (X.S.);1616391011@mail.gxu.cn (S.Y.); wumin@gxu.edu.cn (M.W.)

2 Guangxi Key Laboratory of Clean Pulp & Papermaking and Pollution Control, Guangxi University, Nanning 530004, China

3 Department of Chemical and Paper Engineering, Western Michigan University, Kalamazoo, MI 49008-5200, USA; xiuyu.liu@wmich.edu (X.L.)

4 Department of Pulping and Papermaking Engineering, Guangxi Vocation & Technical Institute of Industry, Nanning 530004, China; jiangyan306@mail.gxu.cn (Y.L.)

* Correspondence: wangsf@gxu.edu.cn; Tel.: +86-159-9434-1859

† These authors contributed equally to the work.

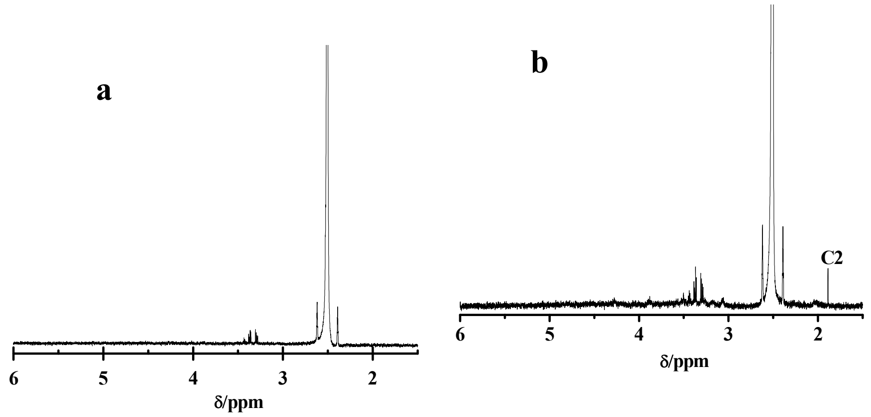

Figure S1. The proton nuclear magnetic resonance (^{1}H-NMR) spectra of (a) NFC and (b) ANFC.

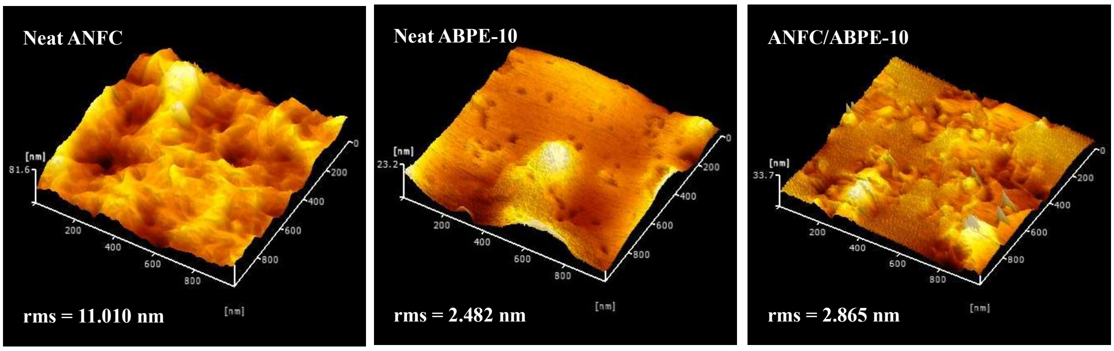

Figure S2. Surface roughness and morphological stability of neat ANFC film， Neat ABPE-10 film， and ANFC/ABPE-10 composite film (68% ANFC).
